# Physical Properties of a Filler-Containing Dental 3D Printing Resin Processed Using Printer Systems with Different Light-Source Wavelengths

**DOI:** 10.3390/ma19194140

**Published:** 2026-09-28

**Authors:** Norihiro Sasamoto, Takashi Horiguchi, Kohei Shintani, Katsushi Okuyama, Yukimichi Tamaki, Tomofumi Sawada

**Affiliations:** 1 Department of Dental Material Science, Division of Oral Functional Science and Rehabilitation, Asahi University School of Dentistry, Hozumi 1851, Mizuho 501-0296, Gifu, Japan; sasamoto@asahi-u.ac.jp (N.S.); guchi@asahi-u.ac.jp (T.H.); kshin_dmd@asahi-u.ac.jp (K.S.); 2 Department of Operative Dentistry, Division of Oral Functional Science and Rehabilitation, Asahi University School of Dentistry, Hozumi 1851, Mizuho 501-0296, Gifu, Japan; katsu@asahi-u.ac.jp; 3 School of Dentistry, Asahi University, Hozumi 1851, Mizuho 501-0296, Gifu, Japan; tamaki@asahi-u.ac.jp

**Keywords:** additive manufacturing, dental 3D printing resin, vat photopolymerization, light-source wavelength, filler-containing resin

## Abstract

**Highlights:**

- Properties of a filler-containing dental resin processed using 405 and 385 nm printer systems were compared.
- Specimens fabricated using the 385 nm printer system showed higher flexural strength and Vickers hardness.
- Color differences in the 385 nm group remained below the clinically acceptable threshold.
- Printer-system characteristics, including light-source wavelength, should be considered in dental 3D printing.

**Abstract:**

This study compared the physical properties and color stability of specimens fabricated from a commercial filler-containing dental 3D printing resin using printer systems equipped with 405 or 385 nm light sources. Specimens were subjected to three-point flexural strength, Vickers hardness, water sorption, solubility, and color stability tests. Color stability was evaluated under dry (A), water-immersed (B), light-shielded (C), and light-exposed (D) conditions, and color differences (ΔE_00_) were calculated using the CIEDE2000 formula. Data were analyzed using Student’s *t*-test or Welch’s *t*-test, as appropriate (α = 0.05). Flexural strength and Vickers hardness were significantly higher, and solubility was significantly lower, in the 385 nm group than in the 405 nm group (*p* < 0.05), whereas water sorption did not differ significantly. The ΔE_00_ values were below the clinically acceptable threshold of 1.77 for all comparisons except A–B in the 405 nm group. The A–B and A–C values were significantly lower in the 385 nm group, whereas the B–C value was significantly higher (*p* < 0.05); no significant difference was observed for C–D. These findings suggest that differences between printer systems, including light-source wavelength, should be considered when fabricating dental prostheses using 3D printing resins.

## 1. Introduction

Additive manufacturing using three-dimensional (3D) printers has attracted increasing attention for fabricating dental crown prostheses owing to advances in digital technology [1,2,3]. In addition to conventional fabrication methods, such as casting, polymerization, and sintering, computer-aided design/computer-aided manufacturing (CAD/CAM) systems are widely used for milling, enabling high-strength, high-toughness ceramics such as zirconia, as well as metals, to be fabricated with high precision. However, the high cost of machinery and material waste during milling remains a challenge [4,5,6,7,8].

In contrast, 3D printers that primarily use light-polymerized resins have the advantage of building and curing only the amount of material required, resulting in minimal material waste, and can be operated at relatively low cost [8]. Therefore, they are expected to become an alternative approach for fabricating crown prostheses. Most resin materials used in clinically available 3D printers to date have had low filler contents and have been reported to exhibit inferior mechanical and chemical properties compared with milled resin blocks [9,10]. Accordingly, their use in the oral cavity has been limited to short-term applications, such as provisional restorations [11,12].

To address these issues, novel 3D printing resins with high filler contents have recently been developed [13]. The presence of filler particles in resin materials can alter light penetration into the resin through light scattering and absorption, suggesting that differences in light-source wavelength may affect polymerization behavior [14,15]. Although 405 nm light sources have been widely used in 3D printers, 385 nm light sources have recently become increasingly available [16,17]. Therefore, comparing the properties of specimens fabricated from filler-containing resins using printer systems with different light-source wavelengths is clinically relevant. However, few studies have examined this wavelength dependence in detail, and the mechanical properties, chemical stability, and color stability of specimens fabricated from filler-containing 3D printing resins using such systems have not yet been fully clarified. It is also well established that various printing conditions, such as layer thickness and build orientation, can affect the properties of objects fabricated using light-polymerized 3D printers [18,19]. Build orientation has been reported to affect flexural strength and fracture behavior, potentially due to differences in interlayer bonding [18,19].

This study aimed to compare the mechanical properties, chemical stability, and color stability of specimens fabricated from a filler-containing 3D printing resin using two commercial printer systems equipped with different light-source wavelengths. The null hypothesis was that there would be no significant differences in these properties between specimens fabricated using the two printer systems.

## 2. Materials and Methods

### 2.1. Materials and 3D Printers Used in This Study

One commercial filler-containing 3D printing resin (SprintRay Ceramic Crown, SprintRay Inc., Los Angeles, CA, USA) and two 3D printer systems with different light-source wavelengths and printing technologies were used (Table 1). Of the two printer systems, one (Pro 55S, SprintRay) uses digital light processing (DLP) technology with a 405 nm LED light source and has a nominal XY resolution of 55 µm, whereas the other (Pro 2, SprintRay) uses Optical Panel technology with a 385 nm UV-A LED light source and has a nominal XY resolution of 35 µm, according to the manufacturer’s specifications.

### 2.2. Specimen Preparation

Two different specimen shapes were prepared based on the Japanese Industrial Standard (JIS T 6517:2011) [20], which corresponds to ISO 10477:2004 [21]. Briefly, rectangular specimens (25 mm in length, 2.1 mm in width, and 2.3 mm in thickness) and disk specimens (15 mm in diameter and 1.3 mm in thickness) were designed using CAD software (Fusion 360 v.2.021538, Autodesk Inc., San Rafael, CA, USA). The specimens were designed with an additional thickness of 0.3 mm to allow for polishing and removal of surface depressions caused by detachment of the support structures. The designs were saved as Standard Tessellation Language (STL) files and converted into printable data using design support software (SprintRay Cloud, SprintRay) (https://dashboard.sprintray.com/, accessed on 24 September 2026). The specimens were fabricated using each printer at a printing angle of 0° relative to the build platform and a layer thickness of 50 μm. The exposure parameters, including irradiance and exposure time per layer, were automatically set by the manufacturer-provided printing profile for the resin. These parameters could not be manually adjusted, and their specific values were not accessible through the printer software. For each test and printer group, the specimens were fabricated in at least three independent printing runs. The specimens were washed with a cleaning solution (3D Medical Clean, Sankyo Pharmaceutical Co., Ltd., Tokyo, Japan) for 30 s and completely dried. They were then placed flat on the curing tray without overlapping and post-cured approximately for 3 min using a post-curing unit (NanoCure, SprintRay) equipped with 365 and 385 nm LEDs and a temperature-controlled curing system. The manufacturer-provided standard preprogrammed material profile for SprintRay Ceramic Crown was used without modification.

### 2.3. Three-Point Flexural Bending Test

The rectangular specimens were polished using waterproof abrasive papers (#600–#1200 grit; Sankyo Rikagaku Co., Ltd., Okegawa, Japan) (*n* = 9 per group). The dimensions (25 ± 2 mm in length, 2 ± 0.1 mm in width, and 2 ± 0.1 mm in thickness) of the specimens were verified using an electronic digital caliper (DIGIMATIC 500-402, Mitutoyo Corp., Kawasaki, Japan). Before testing, the specimens were immersed in distilled water at 37 °C for 24 h.

The three-point flexural bending test was conducted using a universal testing machine (EZ Graph, Shimadzu Corp., Kyoto, Japan) at a crosshead speed of 1 mm/min and a span length of 20 mm. The flexural strength (*Fs*) was calculated using the following formula [20,21]:

*Fs* = 3*Fl*/2*bh*^2^

where F is the maximum load, l is the span length, b is the specimen width, and h is the specimen thickness.

### 2.4. Vickers Hardness Test

The disk specimens were polished and their dimensions were verified (15 ± 1 mm in diameter and 1 ± 0.1 mm in thickness), as described above (*n* = 5 per group). The specimens were then immersed in distilled water at 37 °C for 24 h before testing.

The Vickers hardness test was conducted using a micro-Vickers hardness tester (HMV-1, Shimadzu) under an indentation load of 1.96 N applied for 10 s [20]. Each specimen was measured at three different locations, and the average value was calculated.

### 2.5. Water Sorption and Solubility Test

The disk specimens were used for the water sorption and solubility test (*n* = 5 per group) [20,21]. The specimens were mirror-polished using a 0.3 µm alumina suspension (High Purity Alumina Suspension 0.3 µm, Sankei Co., Ltd., Tokyo, Japan) and a polishing felt.

Each specimen was dried in a desiccator and weighed every 24 h using an electronic balance (AB265-S, Mettler Toledo, Greifensee, Switzerland). When the decrease in mass was less than 0.1 mg over a 24 h period, the mass was recorded as the baseline mass.

For the water sorption test, each specimen was immersed in water at 37 °C for 7 days and then weighed. Water sorption was calculated by dividing the increase in mass after 7 days of water immersion by the volume of each specimen.

After weighing, the specimens were dried again in a desiccator under the same conditions until a constant mass was achieved. The final mass was recorded, and solubility was calculated by dividing the decrease in mass from the baseline mass by the volume of each specimen.

### 2.6. Color Stability Test

The mirror-polished disk specimens were used for the color stability test (*n* = 5 per group). The specimens were classified into four conditions: A (dry), B (water-immersed), C (light-shielded), and D (light-exposed) (Table 2) [20].

The color of each specimen was measured using a spectrophotometric colorimeter (SE7700, Nippon Denshoku Industries Co., Ltd., Tokyo, Japan). The L*, a*, and b* values of each specimen were measured at five different locations. Color differences between conditions (A–B, A–C, B–C, and C–D) were calculated from the measured L*, a*, and b* values using the CIEDE2000 color difference formula (ΔE_00_) and evaluated as an index of color stability [22].

### 2.7. Statistical Analysis

Separate sets of specimens were used for each test. The sample size for each test was determined according to the number of specimens specified in JIS T 6517:2011 [20]. Statistical analyses were performed using the software (BellCurve for Excel, Social Survey Research Information, Tokyo, Japan). The normality and homogeneity of variance of the data were assessed using the Shapiro–Wilk test and Levene’s test, respectively. Based on these assessments, the flexural strength, Vickers hardness, water sorption, solubility, and color stability data were analyzed using Student’s *t*-test or Welch’s *t*-test, as appropriate. The four color-stability comparisons were prespecified based on JIS T 6517:2011. To control the family-wise Type I error rate across these comparisons, the *p*-values were adjusted using the Holm method. Statistical significance was set at 5%.

## 3. Results

### 3.1. Three-Point Flexural Strength

The mean flexural strength values (±standard deviation) were 180.3 ± 2.0 MPa and 185.9 ± 7.0 MPa for the 405 and 385 nm groups, respectively, with a significantly higher value in the 385 nm group (*p* = 0.045) (Figure 1). Regardless of the printer system, all specimens exceeded the minimum requirements of 80 MPa for flexural strength specified in JIS T 6517:2011 [20].

### 3.2. Vickers Hardness (Hv)

The mean Hv values (±standard deviation) were 42.4 ± 3.5 and 48.8 ± 2.1 for the 405 and 385 nm groups, respectively, with a significantly higher value in the 385 nm group (*p* = 0.007) (Figure 2). Regardless of the printer system, all specimens exceeded the minimum required value of 18 specified in JIS T 6517:2011 [20].

### 3.3. Water Sorption and Solubility

The mean water sorption values (±standard deviation) were 15.9 ± 0.3 and 15.6 ± 0.6 for the 405 and 385 nm groups, respectively. No significant difference in water sorption was observed between the specimens fabricated using 405 nm and 385 nm light sources (Figure 3). Regardless of the fabrication condition, all specimens were below the required value of 40 µg/mm^3^ specified in JIS T 6517:2011 [20].

The mean solubility values (± standard deviation) were 0.7 ± 0.1 and 0.5 ± 0.1 for the 405 and 385 nm groups, respectively. Solubility was significantly lower in the specimens fabricated using the 385 nm light source than in those fabricated using the 405 nm light source (*p* = 0.007) (Figure 4). Regardless of the fabrication condition, all specimens were below the required value of 7.5 µg/mm^3^ specified in JIS T 6517:2011 [20].

### 3.4. Color Stability

The ΔE_00_ values between each condition for the specimens are shown in Figure 5. The values were below the clinically acceptable threshold of 1.77 [22] for all specimens, except the A–B comparison in the specimens fabricated using the 405 nm light source.

For the A–B and A–C comparisons, the ΔE_00_ values were significantly lower in the specimens fabricated using the 385 nm light source than in those fabricated using the 405 nm light source (*p* < 0.001). In contrast, the ΔE_00_ values for the B–C comparison were significantly higher in the specimens fabricated using the 385 nm light source than in those fabricated using the 405 nm light source (*p* = 0.035). No significant difference was observed for the C–D comparison.

## 4. Discussion

Additive manufacturing technology is classified into seven processes [23]. Among these processes, vat photopolymerization (VPP) is commonly used to fabricate objects from dental 3D printing resin materials. VPP is further classified into stereolithography (SLA), DLP, and liquid crystal display (LCD) based on the irradiation method and light source [24,25]. In this study, two DLP-based 3D printers were used: a printer with a 405 nm light source (SprintRay Pro 55S) and a printer with a 385 nm light source (SprintRay Pro 2). Specimens fabricated using the 3D printer with the shorter-wavelength 385 nm light source showed higher flexural strength and Vickers hardness and lower solubility. In contrast, no significant differences in water sorption were observed between the two light sources, whereas some significant differences in color stability were observed. Therefore, the null hypothesis was partially rejected.

DLP-based 3D printing enables high-speed and high-precision fabrication and produces little material loss. The finding that the wavelength of the light source affected the mechanical properties and some chemical properties is considered to be attributable to the wavelength used during polymerization of the filler-containing 3D printing resin. In general, the polymerization efficiency of light-polymerized resins is known to depend greatly on how closely the wavelength of the light source matches the absorption characteristics of the photoinitiator [26]. Photoinitiators known to be used in dental light-polymerized resins include camphorquinone (CQ) and 2,4,6-trimethylbenzoyl-diphenylphosphine oxide (TPO). CQ is commonly used in dental composite resins and has an absorption maximum at approximately 470 nm, whereas TPO is used in dental 3D printing resins and has an absorption maximum at approximately 380–420 nm. TPO directly generates radicals upon light irradiation in this wavelength range, thereby efficiently initiating the polymerization reaction [27].

In addition, in filler-containing resin materials, light scattering and absorption by filler particles may affect polymerization behavior [14,15]. However, polymerization efficiency is not determined solely by filler content; rather, it is influenced by multiple factors, including filler particle size and size distribution, the difference in refractive index between the filler and resin matrix, the composition of the resin matrix and photoinitiator, and light irradiation conditions [14,15]. Therefore, for filler-containing resins in which polymerization behavior is affected by multiple factors, using a light source with a wavelength matched to the absorption characteristics of the photoinitiator is considered important for achieving efficient polymerization.

Although both 385 and 405 nm light sources can activate TPO, the 385 nm light source used in this study may have had a wavelength more closely matched to the absorption characteristics of the photoinitiator in this material. This closer spectral match may have resulted in relatively higher excitation efficiency of the photoinitiator even under conditions affected by light scattering from the filler particles. Consequently, polymerization within the resin and between layers may have proceeded more efficiently, resulting in a higher crosslink density of the resin matrix and improved mechanical properties. The flexural strength of the 385 nm group (185.9 ± 7.0 MPa) was numerically higher than the 24 h values reported for seven single-shade universal resin composites (65.34 ± 19.07–170.10 ± 46.37 MPa) [28]. Because the material composition, fabrication method, specimen preparation, and testing conditions differed between the studies, these values cannot be directly compared.

Regarding water sorption, it is known to depend primarily on the type and amount of polar functional groups present in the main and side chains of the monomers constituting the resin [29]. These characteristics are determined by the chemical composition of the material and are not expected to change substantially before and after polymerization. Therefore, the lack of a statistically significant difference between the two light-polymerization wavelengths in this study may be attributed to the identical chemical composition between the two groups. In contrast, the specimens fabricated using the 385 nm light source showed significantly lower solubility than those fabricated using the 405 nm light source. As described above, this finding may be associated with more efficient polymerization and a consequent reduction in the amount of unreacted components; however, the degree of conversion was not directly measured in this study. The water sorption and solubility of the specimens fabricated using the 385 nm light source in this study were lower than those of conventional composite resins [30].

Regarding color stability, the results indicate that the filler-containing 3D printing resin used in this study exhibited relatively high resistance to color change under the tested conditions, as most color differences remained within the clinically acceptable range [22]. The lower ΔE_00_ values observed with the 385 nm light source under some condition comparisons may be attributable to a higher degree of polymerization, which could have increased the crosslink density of the resin matrix and thereby reduced its susceptibility to structural changes.

Finally, the findings suggest that the printer system used may influence the properties of 3D-printed crown prostheses. However, because two different printer systems were compared, the effect of light-source wavelength could not be isolated from other printer-specific factors. Furthermore, because the exact irradiance and exposure time per layer were not disclosed by the manufacturer, the energy dose could not be calculated. Therefore, the findings should be interpreted as differences between the two printer systems rather than as effects attributable solely to light-source wavelength. This study evaluated a single material under limited fabrication conditions. Although comparisons with other filler-containing resins are needed, material–printer compatibility may limit such evaluations. As the degree of conversion and crosslinking density were not directly measured in this study, their contributions to the observed properties could not be confirmed. Further studies should directly evaluate the degree of conversion and crosslinking density and investigate the effects of build orientation, layer thickness, post-curing conditions, and filler content. Long-term durability under thermal cycling and time-dependent color changes should also be evaluated.

## 5. Conclusions

This study compared the physical properties and color stability of specimens fabricated from a filler-containing dental 3D printing resin using different printer systems. The specimens fabricated with the 385 nm light source showed significantly higher flexural strength and Vickers hardness and significantly lower solubility than those fabricated with the 405 nm light source, whereas no significant difference was observed in water sorption. Most color differences were below the clinically acceptable threshold. These findings indicate that the printer system, including its light-source wavelength, may affect the physical properties and color stability of the tested resin.

Further studies using different materials and controlled printing and post-curing conditions are needed to evaluate compressive strength, abrasion resistance, thermal stability, and long-term durability and to clarify the specific influence of light-source wavelength.

## Figures and Tables

**Figure 1:**
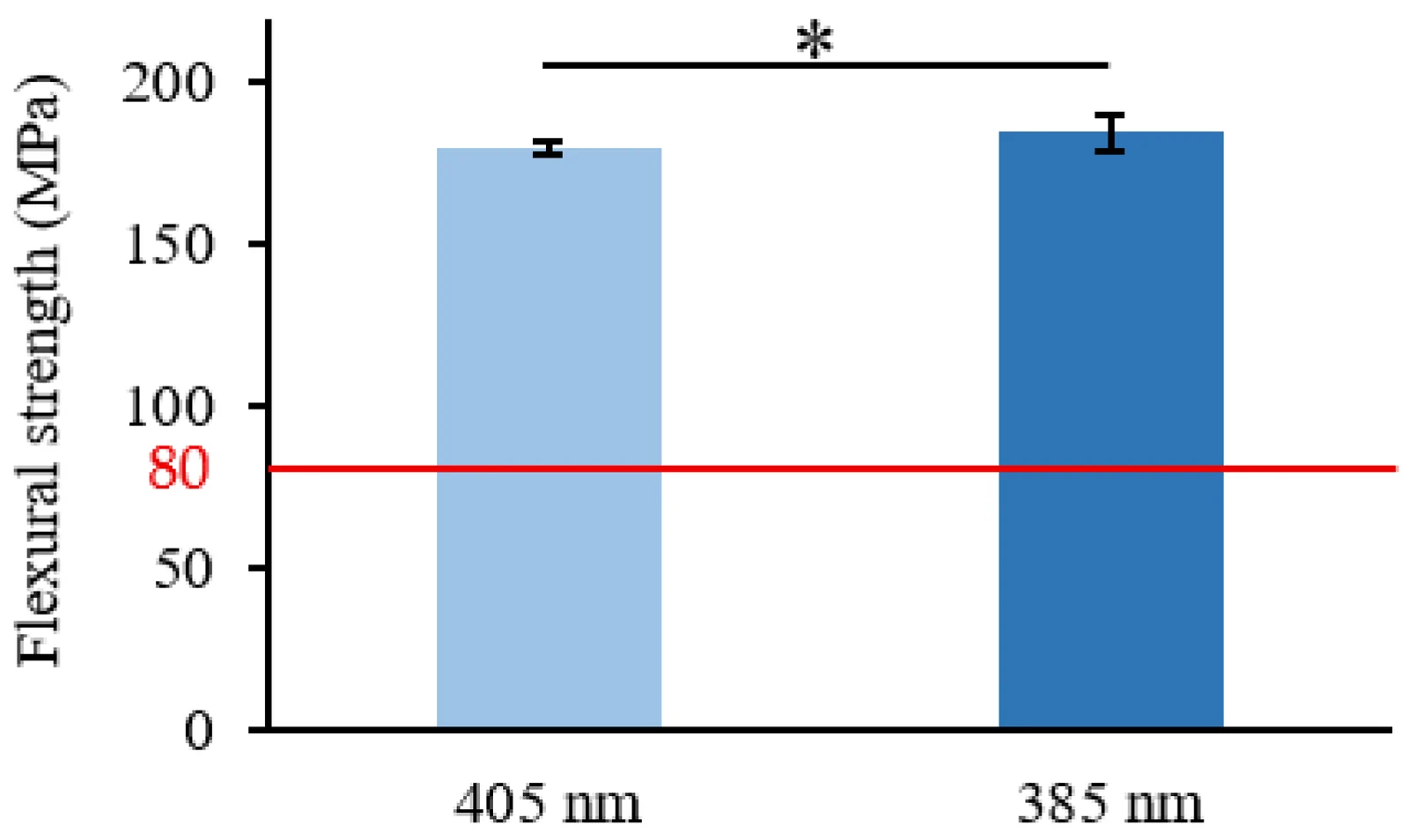
Three-point flexural strength of the specimens fabricated using 405 nm and 385 nm light sources. Values are given as mean and standard deviations. The asterisk indicates a significant difference (*p* < 0.05). The red line indicates the minimum required value of 80 MPa specified in JIS T 6517:2011 [20].

**Figure 2:**
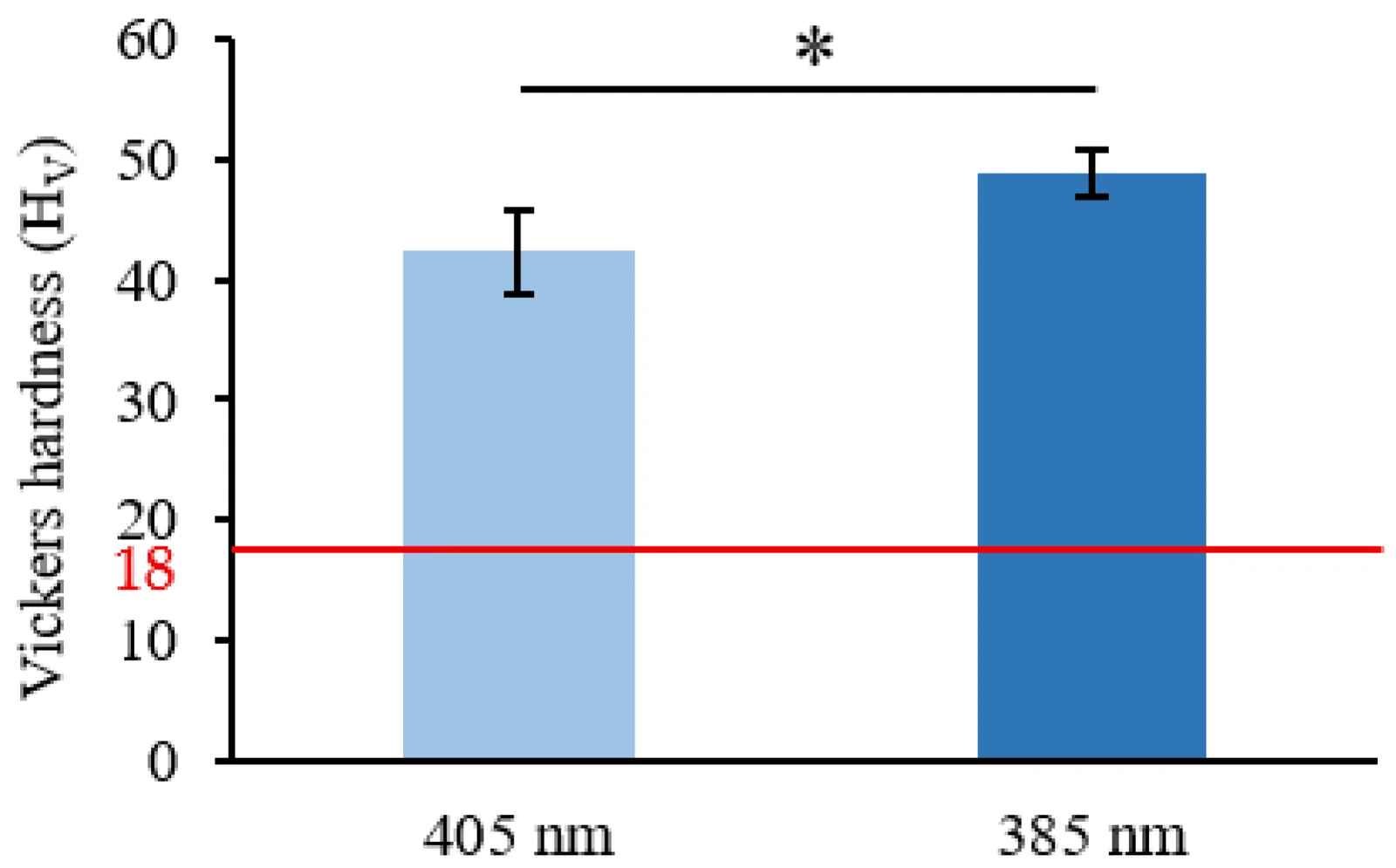
Hv of the specimens fabricated using 405 nm and 385 nm light sources. Values are given as mean and standard deviations. The asterisk indicates a significant difference (*p* < 0.05). The red line indicates the required value of 18 Hv specified in JIS T 6517:2011 [20].

**Figure 3:**
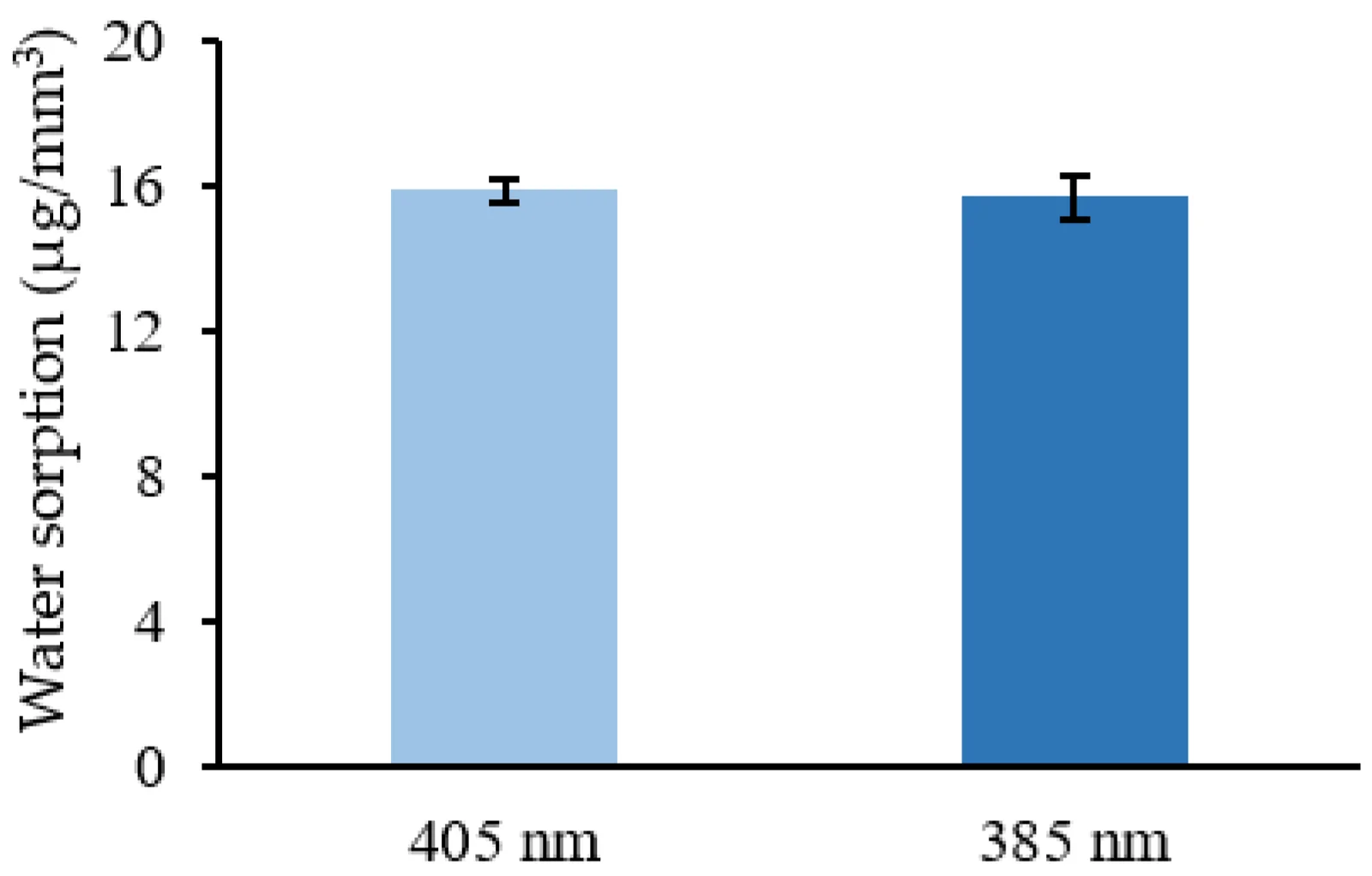
Water sorption of the specimens fabricated using 405 nm and 385 nm light sources. Values are given as mean and standard deviations.

**Figure 4:**
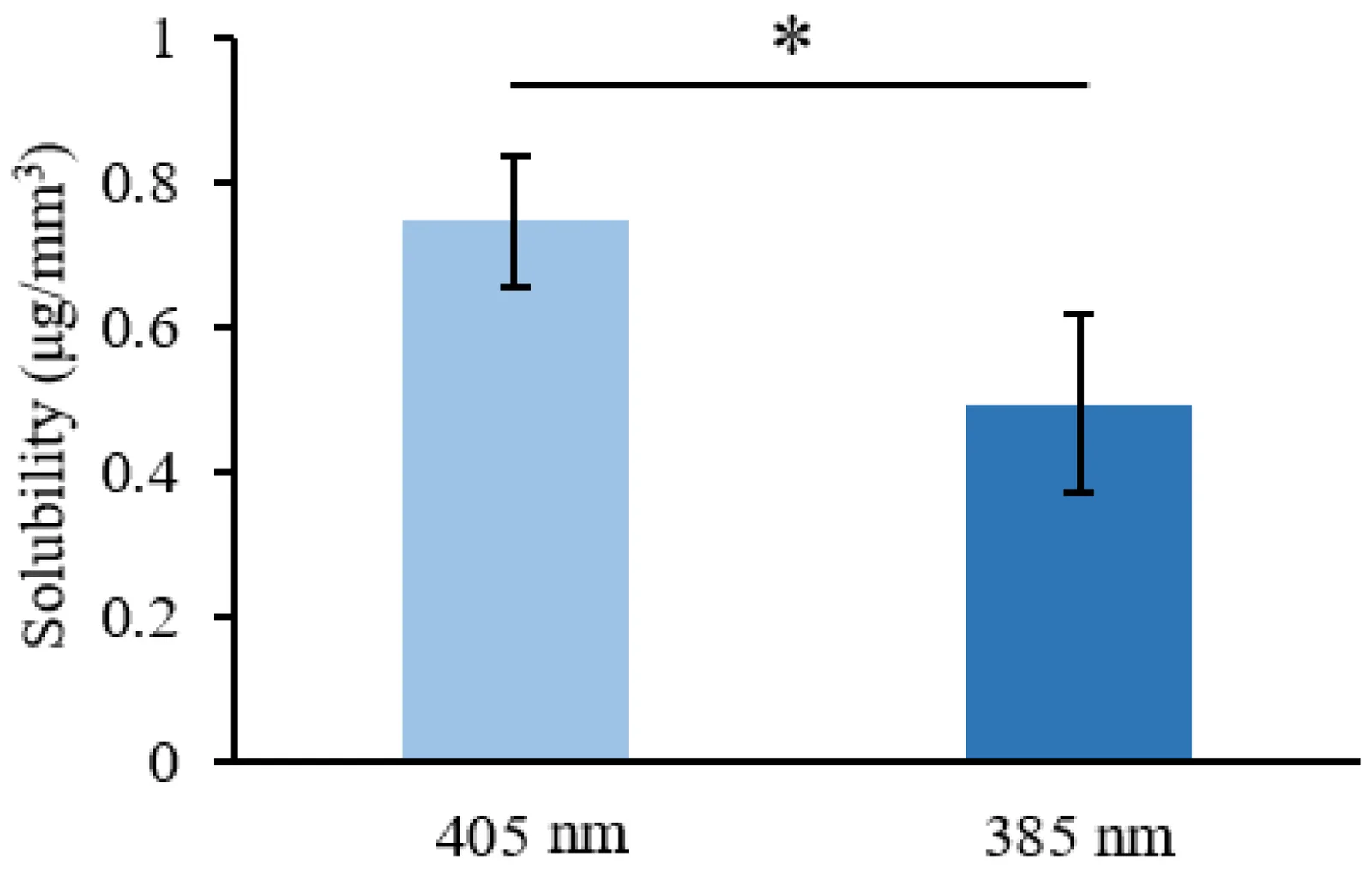
Solubility of the specimens fabricated using 405 nm and 385 nm light sources. Values are given as mean and standard deviations. The asterisk indicates a significant difference (*p* < 0.05).

**Figure 5:**
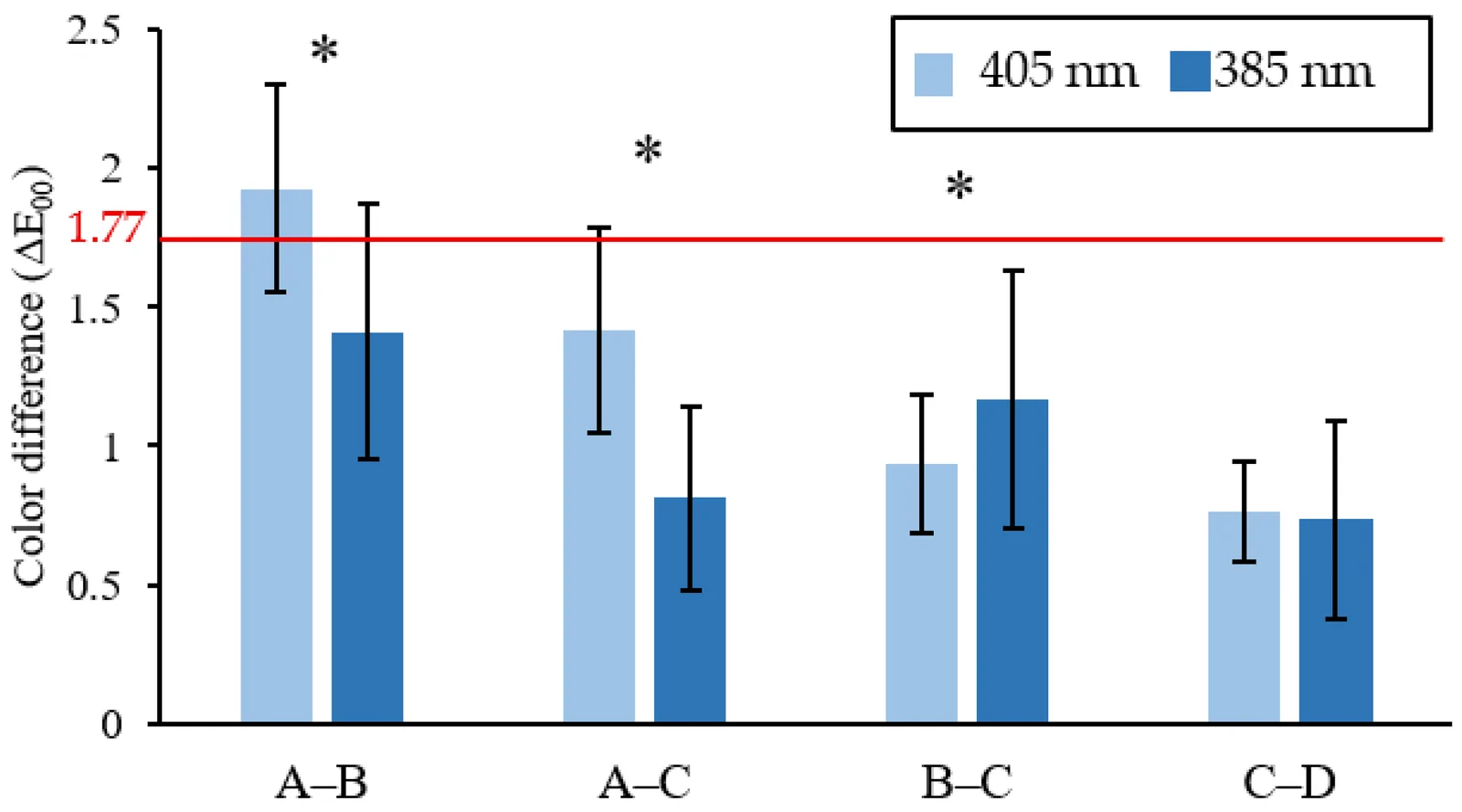
Color differences (ΔE_00_) between each condition for the specimens fabricated using 405 nm and 385 nm light sources. Values are given as mean and standard deviations. The asterisk indicates a significant difference (*p* < 0.05). The red line indicates ΔE_00_ = 1.77, the clinically acceptable threshold [22].

**Table 1:** Materials and 3D printers used in this study.

	Product (Lot)	Composition/Technical Specifications *	Manufacturer
Material	Ceramic crown (S24G033)	Amorphous silica (20–50%), Bisphenol A methacrylate (10–40%), Ytterbium fluoride (10–30%), UDMA (5–15%), TMSPMA (0–2%), TPO (0–2%), Silica dimethyl silylate (0–2%)	SprintRay
3D printer	Pro 55S (PS5P0824A10007)	DLP technology; 405 nm LED light source; nominal XY resolution, 55 µm	SprintRay
	Pro 2 (P23P0224A17496)	Optical Panel technology; 385 nm UV-A LED light source; nominal XY resolution, 35 µm	SprintRay
Cleaning solution	3D Medical Clean (207076)	Ethanol (80–90%), n-propyl alcohol (<10%), isopropanol (<5%)	Sankyo Pharmaceutical
Post-curing unit	NanoCure (PCMA0625A10348)	Temperature-controlled post-curing system; dual-wavelength 365 and 385 nm LEDs	SprintRay

* As disclosed by the manufacturers. DLP: Digital light processing, LED: light-emitting diode, TMSPMA: 3-(Trimethoxysilyl)propyl methacrylate, TPO: 2,4,6-Trimethylbenzoyl diphenylphosphine oxide, UDMA: Urethane dimethacrylate.

**Table 2:** Test conditions for color stability test according to JIS T 6517:2011 [20].

Condition	Description
A (dry)	Stored under dry and dark conditions at 23 °C for 7 days.
B (water-immersed)	Immersed in water at 37 °C in an incubator for 7 days. After immersion, the specimens were removed from the water and blotted with white filter paper.
C (light-shielded) and D (light-exposed)	Stored under dry and dark conditions in an incubator at 37 °C for 24 h. Half of each specimen was covered with metal foil. The specimens were then exposed to direct sunlight for a total of 10 h. After removal of the metal foil, the specimens were placed in an incubator at 37 °C and dried under dark conditions for 5 days. The area covered with metal foil was designated as C, and the uncovered area was designated as D.

## Data Availability

The data presented in this study are openly available in Asahi University at https://asahi-u.repo.nii.ac.jp/ “(URL accessed on 24 September 2026)”.
